# Prevalence of scrub typhus in a tertiary care centre in Telangana, south India

**Published:** 2020-06

**Authors:** Ram Mohan Mylavarapu Venkata Naga Lakshmi, Teja Vijay Dharma, Sukanya Sudhaharan, Subbalaxmi Malladi Venkata Surya, Rajkiran Emmadi, Satyanarayana Raju Yadati, Nageswara Rao Modugu, Aparna Jyotsna

**Affiliations:** 1 Department of Microbiology, Nizam’s Institute of Medical Sciences, Punjagutta, Hyderabad, Telangana, India; 2 Department of General Medicine, Nizam’s Institute of Medical Sciences, Punjagutta, Hyderabad, Telangana, India

**Keywords:** Scrub typhus, Immunochromatographic test, Acute undifferentiated fever, Enzyme linked immunosorbent assay

## Abstract

**Background and Objectives:**

Scrub typhus is re-emerging as an important cause of acute undifferentiated fever in the last decade from various parts of India. Complexity in performing the “gold standard” immunofluorescent assay and the unreliable nature of Weil Felix test often results in delayed or misdiagnosis in a majority of cases. The present study seeks to integrate the results of rapid diagnostic tests, clinical and laboratory features to aid the diagnosis and management of scrub typhus patients.

**Materials and Methods:**

A total of 645 serum samples with suspected scrub typhus sent to the Department of Microbiology were included in the study. Scrub typhus was tested by rapid immunochromatographic test (SD Diagnostics) and IgM ELISA (Inbios International, USA). Clinical features, laboratory parameters and final outcome were analysed from the clinical records of positive patients.

**Results:**

Scrub typhus was diagnosed in 13.7% of patients and majority of them were observed in the month of August. 58.6% of scrub typhus patients presented with fever of one to two weeks duration. Eschar was documented in 13.7% of patients and 24% of patients gave a history of working outdoors or exposure to vegetation. All the patients responded to Doxycycline treatment and there was no mortality.

**Conclusion:**

High index of suspicion for scrub typhus is necessary in febrile patients not responding to conventional antibiotics especially during outbreak situations. Rapid immunochromatographic tests with excellent specificity and acceptable sensitivity can be used as potential point of care tests for quick diagnosis of scrub typhus especially in delayed presentation.

## INTRODUCTION

Scrub typhus is a serious public health problem in the Asia-Pacific region threatening one billion people globally and causing illness in one million people each year (1). It is caused by the arthropod-borne Gram-negative obligate intracellular bacillus *Orientia tsutsugamushi* and is an important cause of acute undifferentiated febrile illness (AUFI).

It is a re-emerging disease with several outbreaks reported from various parts of India in the last decade (2–6). Misdiagnosis and under diagnosis of this important cause of acute undifferentiated fever is common due to lack of reliable diagnostic tests and the nonspecific nature of symptoms, especially in the absence of characteristic eschar. As scrub typhus has no specific clinical manifestations, it is important to bring awareness among the clinicians about the clinical presentations, laboratory parameters and confirmatory diagnostic tests.

The present study was undertaken to estimate the prevalence of scrub typhus during the monsoon and immediate post monsoon in the year 2018 and analyse the clinical and laboratory profile of scrub typhus patients.

## MATERIALS AND METHODS

This was a retrospective observational study conducted in Nizam’s Institute of Medical Sciences, a tertiary care hospital in Hyderabad, Telangana, South India, between July to October 2018. Of the total 932 patients with acute pyrexia during the study period, serum samples from 645 clinically suspected scrub typhus patients were included in the study.

Scrub typhus was diagnosed by SD Bioline Tsutsugamushi, a solid phase immunochromatographic (ICT) assay which detects IgG, IgM or IgA antibodies to scrub typhus. IgM ELISA was performed by using scrub typhus Detect IgM ELISA (InBios International Inc., Seattle, WA, USA). A cut off Optical density (OD) >0.5 was considered positive in IgM ELISA. Patient records of samples positive by IgM ELISA and ICT were evaluated for clinical features, laboratory parameters and outcome. Statistical analysis was performed using the Graph Pad prism statistical software; categorical variables were compared using Fisher’ Test. p value less than 0.05 was considered significant.

## RESULTS

Scrub typhus was clinically suspected in 69% (645/932) of patients with acute pyrexia and 13.7% (89/645) of them were positive by both Scrub typhus rapid test and IgM ELISA.

**Fig. 1.**
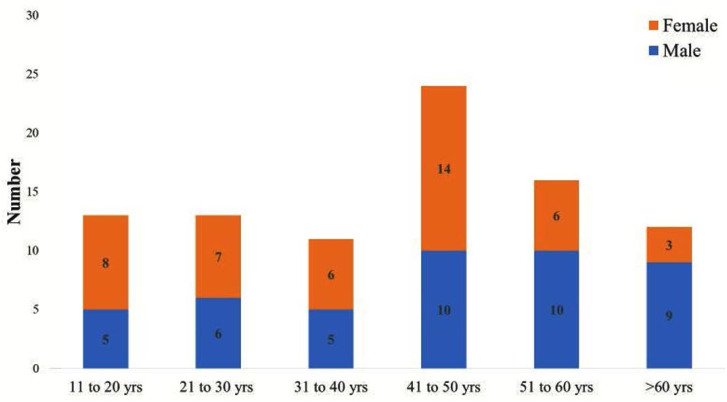
Age and gender distribution of Scrub typhus patients

There was no significant gender difference in the distribution of scrub typhus cases and 48/89 (54%) of them were in the age group of 20–50 years (Fig. 1). Peak incidence of scrub typhus was observed in August and decreased thereafter.

**Fig. 2.**
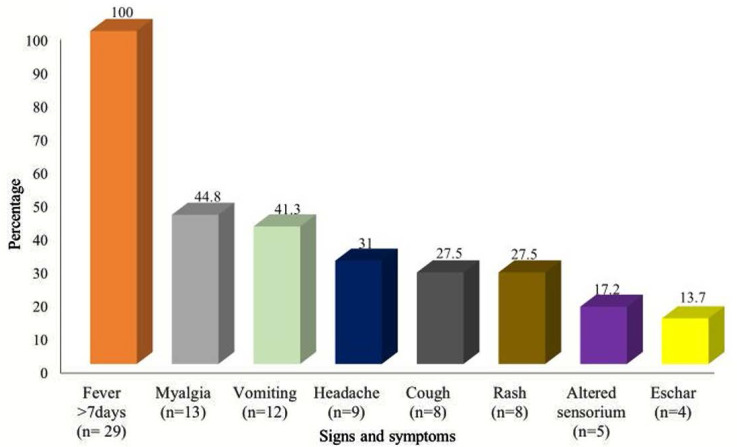
Signs and symptoms in Scrub typhus patients

Clinical records of the twenty-nine scrub typhus patients were available for analysis. Average duration of fever was 8.3 days. Majority of the patients (17/29; 58.6%) presented with 7–14 days of fever and 10.3% (3/29) of them had prolonged pyrexia beyond 2 weeks. Eschar was present in 13.7% of patients (Fig. 2). Seven of the 29 patients (24.1%) were either agricultural workers or had history of exposure to vegetation.

Markers of severe disease include elevated aspartate amino transferase (AST), urine albumin and serum creatinine and were seen in 82.7%, 31% and 20.6% patients respectively. Complications were seen in 8 patients– pneumonitis in 3 patients; shock, renal failure, acute respiratory distress syndrome (ARDS) and myocarditis in one patient each. One patient had both pneumonitis and shock. Clinical scoring system suggested by Jung et al. (7) was used to estimate the probability of disease; age >65 years, presence of eschar and onset of illness during an outbreak of scrub typhus carried 2 points each and recent history of field work or myalgia were awarded 1 point each. 20/29 (68.9%) of patients had a score of ≥3 which correlates with 100 % sensitivity for scrub typhus. Defervescence was achieved in all the patients within 2 days of doxycycline treatment and there was no mortality.

## DISCUSSION

Acute undifferentiated fevers are very common in tropical countries like India during monsoon and post monsoon period as they are conducive for mosquitoes and mites to breed and flourish. High prevalence of infective vectors, increased contact between humans and vectors, temperature, humidity, prolonged rains, rich growth of vegetation favour disease transmission. The similarity in clinical presentation and diverse etiological agents further complicate the diagnosis and treatment of AUFI.

The prevalence of scrub typhus varies from 0–8% to 60% in different countries (8). The prevalence of scrub typhus in the present study was 13.7% compared to other Indian studies which range from 13 to 63% (9–11).

Most of the cases were seen in 20 to 50 year age group with no gender predominance similar to observations made by Sivarajan et al. (9) while a slight male preponderance was reported by two studies from South India (11, 12). Bridging of the difference in the gender gap could be a reflection of the socio-cultural change with increasing number of women working outdoors. Peak incidence of scrub typhus was seen in August in the present study followed by a decline in the succeeding months. In south India, scrub-typhus cases occur mostly in the cooler months (August–January), while in Southeast Asia, scrub-typhus cases are highest in July–November (13).

Fever was the most common clinical presentation and 58.6% of patients presented with 1–2 weeks of fever as observed in several studies (4, 10–12, 14, 15). As complications are more likely to occur after the first week of illness, a high index of suspicion for scrub typhus is needed for prompt diagnosis, treatment and subsequent reduction in mortality in such patients. The presence of diagnostic eschar in scrub typhus is variable; it is more easily found on Caucasian and East Asian patients than on dark skinned South Asian patients (16). Eschar was documented in 13.1% patients in our study compared 7% to 97% in various studies (17). A prospective study on 161 patients in Northern Thailand to find the true accuracy of diagnostic tests for scrub typhus concluded that a combination of ICT and presence of eschars has good specificity and can be used in resource-poor situations as point-of-care diagnostic test (18).

The utility of Indirect Immunofluorescence assay (IFA) as a gold standard for diagnosis of scrub typhus is limited by the requirement of standard slides, paired sera and subjective interpretation. Weil-Felix test is widely used but lacks sensitivity and specificity (19). Though IgM ELISA has been reported to have good sensitivity and specificity (20), it requires pooling of samples and takes 3 to 4 hours for the complete procedure. Rapid ICT has been reported to have very good specificity and sensitivity. Evaluation of the SD Bioline ICT in Thailand patients in 2012 has shown that it is more sensitive than IFA with specificity as high as 98.4% in diagnosing acute phase samples (21). SD Bioline ICT has been reported to have high sensitivity (99%), specificity (96%) and serological agreement (97.5%) with immunofluorescent assay (22). A correlation of 97% between IgM ELISA and SD Bioline Tsutsugamushi rapid diagnostic test was reported in a study from South India similar to our findings (11).

There was no mortality in the present study compared to 4.5% mortality from a similar study in our institute in 2014. This could be attributed to the increased awareness of the diverse clinical presentation of scrub typhus among the clinicians, early diagnosis and treatment. Delayed treatment in patients with scrub typhus increases morbidity and mortality (23–25). A study evaluating risk factors for ARDS in scrub typhus patients established that prolonged untreated fever was significantly associated with ARDS (26).

Majority of the patients in developing countries with AUFI reach tertiary care hospital in the 1st week to 10 days of illness by which time levels of IgM antibodies would have reached a threshold of detection by rapid ICT’s. A study to assess the diagnostic utility of ICT for early rapid diagnosis of scrub typhus in Korean patients found that ICT was positive in 63.6% of scrub typhus cases with ≤5 days fever and increased to 77.8% and 92.3% in fevers of 6–10 days and ≥11 days duration respectively (27). Hence rapid ICT’s can be used as potential point of care tests for delivering positive results quickly to physicians to initiate specific treatment without any delay.

In conclusion, clinical and diagnostic workup for Scrub typhus is warranted in all undifferentiated fevers of more than 1 week duration not responding to conventional empirical antibiotics. As results of confirmatory tests are not instantly available, a combination of clinical features, laboratory parameters and a positive ICT result would be useful in early initiation of specific therapy in patients with scrub typhus.
